# Facilitators and barriers in the diagnostic process of vulvovaginal complaints (vulvodynia) in general practice: a qualitative study

**DOI:** 10.1080/13814788.2017.1420774

**Published:** 2018-01-23

**Authors:** Peter Leusink, Doreth Teunissen, Peter L. Lucassen, Ellen T. Laan, Antoine L. Lagro-Janssen

**Affiliations:** ^a^ Department of Primary and Community Care, Unit Gender & Women’s Health, Radboud University Medical Centre Nijmegen the Netherlands; ^b^ Department of Sexology and Psychosomatic Obstetrics and Gynaecology, Academic Medical Center, University of Amsterdam Amsterdam the Netherlands

**Keywords:** (MeSH), general practice, vulvodynia, clinical decision-making, differential diagnosis, uncertainty

## Abstract

**Background:** The gap between the relatively high prevalence of provoked vulvodynia (PVD) in the general population and the low incidence in primary care can partly be explained by physicians’ lack of knowledge about the assessment and management of PVD.

**Objectives:** To recognize barriers and facilitators of GPs in the diagnostic process of women presenting with recurrent vulvovaginal complaints.

**Methods:** A qualitative focus group study in 17 Dutch GPs, five men and 12 women. An interview guide, based on the scientific literature and the expertise of the researchers, including a vignette of a patient, was used to direct the discussion between the GPs. The interviews were audiotaped and transcribed verbatim. A systematic text analysis of the transcripts was performed after data saturation was reached.

**Results:** Analysis of the interviews generated three major themes: Identifying and discussing sexual complaints, importance of gender in professional experience, and coping with professional uncertainty. Within these themes, the reluctance regarding sexual complaints, male gender, negative emotional responses when faced with professional uncertainty, as well as lack of education were barriers to the diagnostic process and management of PVD. Female gender and understanding that patients can profit from enquiring about sexual health issues were found to be facilitating factors.

**Conclusions:** To improve the care for women with PVD, attitude and skills of GPs regarding taking a sexual history and performing a vulvovaginal examination should be addressed, as well as GPs’ coping strategies regarding their professional uncertainty.

Key Messages

- Female and certainly male GPs should be better supported in the development of attitudes and skills regarding taking a sexual history in women and performing a vulvovaginal examination.
- GPs should be trained to address vulvodynia like other medically unexplained physical symptoms, as GPs face the same professional uncertainty about it.

## Introduction

Vulvodynia is a common disorder in the general population with a point prevalence of 8% and a lifetime prevalence of 25% [1,2]. Vulvodynia is defined as ‘vulvar pain of at least three months duration, without clear identifiable cause, which may have potential associated factors [3]. The following descriptors are used to specify the characteristics of vulvodynia: Localized (e.g., vestibulodynia, clitorodynia) or generalized or mixed; provoked (e.g., insertional, contact) or spontaneous or mixed; onset (primary or secondary); and temporal pattern (intermittent, persistent, constant, immediate, delayed) [3]. As provoked vulvodynia (PVD) is the most prevalent and studied clinical presentation of vulvodynia, with many implications on an individual and relational level, the focus of this article will be on PVD.

Studies show that about 50% of women with PVD never sought help and of those who did, more than 50% received no diagnosis [1,2]. Only 5.7% of those who sought help were diagnosed with vulvodynia [1]. Accordingly, in Dutch family practices the registered incidence of painful intercourse, an essential symptom of PVD, is very low, about 0.1% [4]. More precise epidemiological findings of PVD in primary care are rare since PVD is not included in the International Classification of Primary Care (ICPC).

The gap between the relatively high prevalence of PVD in the general population and the low incidence in primary care can be explained by patient factors as well as by physician factors [5]. As was demonstrated in some studies, physicians lack knowledge about the assessment and management of PVD [6–8]. In one population-based study, 60% of women with PVD consulted three or more doctors, many of whom were unable to provide a clear diagnosis [6]. In a qualitative, in-depth interview study, women with PVD consulted between three and 15 different doctors and often had endured months of incorrect treatment, most notably for vulvovaginal candidiasis [7]. Furthermore, a survey among junior gynaecologists showed that most of them did not receive any basic training about the condition, even after having reached the final stages of specialism training [8]. Possibly, physicians’ lack of competence to recognize and to diagnose PVD maintains the negative beliefs of women regarding the benefit of seeking help.

General practitioners (GPs) deal with vulvovaginal complaints regularly and the diagnostic process proves to be complicated [9,10]. In a retrospective cohort analysis, we found that in a general practice population of women between 15 and 50 years, symptoms suggestive of PVD were strongly associated with the diagnosis of vulvovaginal candidiasis (OR: 4–7) [11]. Moreover, in women, in general practice who presented recurrent and persistent vulvar complaints diagnosed as vulvovaginal candidiasis, concurrent symptoms, such as dyspareunia, functional syndromes, micturition symptoms and psychological conditions, may point to the diagnosis of PVD [12]. GPs might reconsider their diagnostics and management when women present with recurrent and persistent vulvovaginal complaints.

To improve the recognition of PVD, it is important to know more precisely, which barriers and facilitators are affecting the diagnostic process for GPs who are confronted with recurrent and persistent vulvovaginal complaints. The present article reports on a qualitative focus group study in Dutch GPs investigating their diagnostic considerations when consulted by women with vulvovaginal complaints.

## Methods

### Study design

Focus group interviews were carried out among Dutch GPs during the first half of 2016. Qualitative data were collected to obtain in-depth insight into the clinical decision-making process concerning vulvovaginal complaints. Group interviews rather than interviews with individual GPs were carried out because we expected that the exchange of ideas and experiences between focus group members would generate more relevant information than individual interviews [13].

### Participants

Participants were recruited among 400 randomly selected GPs working in the eastern and middle region of the Netherlands through an invitational letter. Non-responders were contacted two weeks later by e-mail or telephone. Finally, of the 20 GPs who responded, three could not attend the scheduled focus group meeting; 17 participated in four focus groups, five men and 12 women. Each group consisted of four to five participants; three groups contained both men and women, one focus group contained five female GPs. The reason for the low participation rate is not known, as non-response was not investigated. Characteristics of participants are given in Table 1.

**Table 1.** Characteristics of the participants of the focus groups (*n* = 17).

	*n*
Sex	
Female	12
Age	
<45 years	4
45–55 years	8
>55 years	5
Working years as GP	
<10 years	4
10–25 years	9
>25 years	4
Practice type	
Solo	2
Duo	4
Group	11
Active role in education	
Yes	14
Specific interest vulvovaginal complaints	
Yes	11

All GPs signed an informed consent form before participating. Anonymity and confidentiality were ensured. According to Dutch legislation, no approval of a medical ethics committee was required for the interviews. Participants’ travel expenses were reimbursed.

### Focus group interviews and data collection

A certified sexologist (and former GP) moderated two focus groups. Due to practical circumstances, another GP moderated the other two focus groups. The moderators were not personally or professionally related to the participants. An interview guide was used to direct the discussion and was based on the scientific literature and the expertise of the researchers. The emphasis in this guide was placed on the thoughts and feelings of GPs regarding taking a sexual history and performing an examination of vulva/vagina during their last consultation with a woman with vulvovaginal complaints and when confronted with a vignette. This vignette shows a woman consulting the GP the third time in four months (including once by telephone) with ‘vulvar irritation without discharge.’ PVD was not suggested as a diagnosis. The basis of the conversation was the GP's diagnostic process. The focus groups lasted 90 min and were audiotaped and transcribed verbatim.

One author (PLe) and a medical student observed all groups and made field notes of non-verbal communication and potentially remarkable observations.

### Data Analysis

The principal author (PLe) and a medical student independently performed a systematic analysis of transcripts, with one central question in mind: ‘Which facilitators and barriers are observed regarding the diagnosis of vulvovaginal complaints?’ After analysing two group interviews, the codes were discussed with the supervising committee, and some small adjustments in the topic list were made. Particularly, GPs were specifically invited for discussing how they bring up sexual issues with their patients, as they were reluctant to discuss this issue. With this revised topic list we conducted the last two focus groups. After analysing the fourth focus group saturation was reached. A statement of a GP was considered important when it was recognized by others and was followed by discussion. Codes referring to the same content were grouped into categories and subsequently in themes. We applied all consolidated criteria for reporting qualitative research (COREQ-criteria), with the exception of the criterion for returning the transcript to the participants for comment [14]. The qualitative research software package ATLAS-ti (Scientific Software Development GmbH, Berlin, Germany) was used to assist with registering, searching and coding the data. Quotes, which illustrate the main results, are presented (Box 1, 2, 3) and translated from Dutch to English by a bilingual speaker.

Box 1. Identifying and discussing sexual complaints.

**Table:** 

Reluctance taking a sexual history	‘… so in that case [wondering if someone might have a STD] you can focus on it [sexual behaviour] and even then, let’s say, you would not take a thorough sexual history. It’s more like … how sure do you want to be whether someone might have a chlamydia.’ (F4, FG4, 45–55 years) ‘I think, especially when it comes to peers, that a patient seems to find it uncomfortable. This might be an assumption of mine but I have the impression that the discomfort is mutual.’ (M1, FG2, <45 years) ‘With whom you feel less at ease?’ (Moderator) ‘With women of my own age.’ (M1, FG2, <45 years) ‘And in what way does it affect your consultation?’ (Moderator) ‘That I postpone talking about it [sexuality], until I have exceeded all my questions and still don’t know what it’s going on.’ (M1, FG2, <45 years)
Embarrassment	‘What do you want to recommend to a GP trainee regarding the diagnosis of vulvovaginal complaints?’ (Moderator) ‘Do not hesitate to perform a vulvar examination.’ (M1, FG3, 45–55 years) ‘This applies to a male trainee as well?’ (Moderator) ‘Especially to a male trainee!’ (M1, FG3, 45–55 years) ‘Somehow I have the feeling that it is none of my business, she probably thinks I’m a curious guy.’ (M1, FG2, <45 years) ‘No, I didn’t ask, and eh … well, a bit naïve maybe, but I thought, eh … her husband died 5–6 years ago, so… it didn’t cross my mind.’ (M1, FG3, 45–55 years)
Benefit of discussing sexual issues	‘What I found very instructive this afternoon, is that bringing up sexual issues, is not bad at all. Actually, women are very happy about it when it is discussed.’ (M1, FG2, <45 years) ‘I think it’s funny that you [remark directed towards a male GP] say you have difficulties with this, the sexual history, maybe it’s because I’m a woman that I think of it as usual questions.’ (F2, FG2, <45 years)

F: female GP; M: male GP; FG: focus group; all followed by a unique number.

Box 2. Importance of gender in professional experience.

**Table:** 

Routine	‘When I started as a GP, I was the only woman in the group, so automatically you will get some more experience.’ (F2, FG1, 45–55 years) ‘I am the only man with three female colleagues and now the situation is such that … eh … I manage the complaints related to the male lower urinary tract and … eh … the women insert IUDs and manage vulvovaginal complaints’ (M1, FG3, 45–55 years)
Recognition	‘I understood her worries, and … eh …I experienced the same as A. [another female GP]: that it is not complicated to perform a gynaecological examination or to ask about it [referring to sexuality].’ (F1, FG3, > 55 years) ‘When my attention is completely focussed on the patient, the complaint or the story, I am much more successful in filtering out the things that are necessary to ask more specific questions.’ (F2, FG1, 45–55 years)
Curiosity	‘So I thought, look, a puzzle. This is something we have to solve, so yes, I want to know more details and make a plan. It feels like a challenge.’ (F2, FG3, <45 years) ‘I tried to tickle her a bit [in taking responsibility in finding a solution]. I wonder if she will succeed.’ (F3, FG1, 45–55 years) ‘What do you want to recommend to a GP trainee regarding the diagnosis of vulvovaginal complaints?’ (Moderator) ‘The word curiosity is coming into my mind. [.]. Curious about behaviour, about ideas, about meaning. [.].’ (F1, FG1, 45–55 years) ‘How does curiosity help you, regarding vulvovaginal complaints?’ (Moderator) F1, FG1, 45–55 years: ‘To get a clear picture of the problem.’
Uncertainty	‘Because I see less [referring to vulvovaginal complaints], the less you do something, the less efficient you become at it.’ (M1, FG1, 45–55 years) [When performing a gynaecological examination] ‘In women of my own age, the reluctance [referring to his own reluctance] is the highest. […] When the complaints vanish on trial by treating with miconazole, well, than I can postpone that part of the consultation, that’s the way it goes, often.’ (M1, FG2, <45 years)

F: female GP; M: male GP; FG: focus group; all followed by a unique number.

Box 3. Coping with professional uncertainty.

**Table:** 

Overwhelmed by negative emotions	‘It makes me think—help, what do I have to do next? What is the matter? Now and then I find that difficult.’ (F5, FG4, 45–55 years) ‘It is a troublesome complaint which makes me think, eh…, what can I add to this. It feels like, eh … the knowledge that I can’t do anything.’ (F4, FG4, 45–55 years) ‘Often they want to be referred to a gynaecologist, and often you feel obliged to do so. Actually, you are dealing with MUPS.’ (F2, FG1, 45–55 years) ‘You find yourself with your back against the wall relatively quickly, when you think you asked every question and looked at everything.’ (M1, FG2, <45 years)
Problem focused coping	‘Is there any other way we need to confirm this, or are there additional STD tests we need to do?’ (F5, FG4, 45–55 years) ‘Now I would investigate even better. [.]. First of all I would like to exclude the somatic component.’ (F2, FG1, 45–55 years)
Postponing or avoiding a referral	‘I don’t have any reason to refer. I would not know to whom and what my question would be. I think I can handle it myself.’ (F1, FG1, 45–55 years) ‘I would not know to whom I should refer. And firstly, I want to clarify … eh what am I really thinking of?’ (F2, FG1, 45–55 years) ‘It would be very meaningful to understand what is behind that complaint. So this would be a next step before a gynaecologist is being involved.’ (M1, FG2, <45 years)
Referred reluctantly	‘It doesn’t happen many times, but when I have the feeling that whatever I propose hasn’t been taken seriously by the patient, then … eh … then I do the same, then I let it go too.’ (F1, FG3, >55 years) ‘Yes, me too.’ (M1, FG3, 45–55 years) ‘Yes.’ (M2, FG3, >55 years) ‘So you mean, diagnostic uncertainty?’ (Moderator) ‘Yes, that you are not able to …eh … communicate this adequately to the patient.’ (F1, FG3, >55 years) ‘This feeling then leads to a referral?’ (Moderator) ‘Yes, this can be a reason sometimes, yes, definitely.’ (F1, FG3, >55 years)

F: female GP; M: male GP; FG: focus group; all followed by a unique number.

## Results

Analysis of the interviews generated three major themes: Identifying and discussing sexual complaints, importance of gender in professional experience, and coping with professional uncertainty.

### Identifying and discussing sexual complaints

Most GPs were found to have difficulties in enquiring about sexual behaviour, unless it was related to the assessment of the likelihood of a sexually transmitted disease (STD) (Box 1). Outside the context of STDs, GPs were reluctant to take a sexual history. In particular, male GPs mentioned hesitance regarding female patients of their age and mentioned that this hesitancy to take a sexual history and to perform a vulvovaginal examination applies more to themselves than female GPs.

Some male GPs and no female GPs were afraid to be seen as overly curious, or felt embarrassed or noticed a not distinct feeling of tension when enquiring about sexual issues. Some male GPs feared to be considered interfering in patients’ private life or to be disqualified by the patient, even when private or sexual issues might be relevant for the diagnosis.

A facilitating factor was the knowledge that patients could profit from being asked about sexual issues. Some male GPs were grateful for the information provided by their female colleagues—those women do expect professional attention to sexual health issues.

### Importance of gender in professional experience

Female GPs mentioned that they see more women with vulvovaginal complaints than their male colleagues, and that therefore they may have more routine knowledge and skills regarding these issues (Box 2). Also, some female GPs mentioned that identifying themselves with the women, in the sense that they more easily could recognize the problem, proved a facilitating factor in the diagnostic process.

Finally, a sense of curiosity and challenge in searching for a solution was expressed by some female GPs, whereas male GPs never did so. The latter mentioned seeing fewer women with vulvovaginal complaints than their female colleagues did. Therefore, male GPs described that they were less experienced and trained in gynaecological complaints, causing them to feel insecure about assessing sexuality and performing a gynaecological examination, for example, leading to avoiding or postponing an examination, thereby maintaining their lack of experience.

### Coping with professional uncertainty

Confronted with a vignette of a patient with recurrent vulvovaginal symptoms (consulting the GP for the third time after four months), for whom standard therapeutic management was not successful, GPs reacted with negative emotions like helplessness, discomfort, incompetence, and frustration (Box 3). GPs compared their emotions to their reactions when confronted with other physical complaints that cannot be explained somatically (MUPS, medically unexplained physical symptoms). Because of this, they rarely considered the problem presented in the vignette as a challenge to explore further.

There were three main strategies for coping with this professional uncertainty. One strategy (problem-focused) was to start over from the beginning (examinations, tests) to find out whether anything has been overlooked. A second strategy was postponing or avoiding a referral to another professional until the GPs understood the reason why this referral would be necessary. Thirdly, patients were reluctantly referred because GPs did not feel they had any control or influence over the patient.

Regarding PVD, GPs experienced a lack of education and were not acquainted with this diagnosis. In fact, only one GP spontaneously mentioned PVD in the differential diagnosis.

## Discussion

### Main findings

Three themes appeared to best define facilitators and barriers in the diagnostic process of vulvodynia in general practice.

First of all, within the theme ‘identifying and discussing sexual complaints’, a significant barrier was male gender of the GP. Although all GPs were reluctant to assess sexual complaints, female GPs were less reluctant to take a sexual history and to perform a vulvovaginal examination than male GPs. Other barriers were feelings of embarrassment and uncertainty of the male GPs about whether women would appreciate their professional involvement in sexual health issues.

Concerning the second theme, ‘importance of gender in professional experience’, female gender proved to be an important facilitator. Being a woman appeared to facilitate female GPs in recognizing the burden of their female patients and to improve their expertise as a result of more frequent management of women with vulvovaginal complaints, and of feeling a greater sense of curiosity and challenge in finding a solution. Finally, both male and female GPs expressed uncertainty about their professional expertise when confronted with recurrent vulvovaginal complaints.

Within the theme ‘coping with professional uncertainty’, lack of knowledge and feelings of incompetence were barriers in coping professionally with vulvovaginal symptoms that may be indicative of PVD, a condition GPs considered an example of MUPS. GPs showed three strategies in dealing with this professional uncertainty: starting over diagnostics from the beginning, postponing a referral or referring with reluctance. No facilitators were found within this theme.

### Strengths and limitations

The strength of this study is its reporting of findings that were obtained because of the interaction between GPs, shown by the mutual recognition in identifying sexual complaints and coping with professional uncertainty. Especially the gender issue was raised as a result of the interaction between male and female GPs. Also, to our knowledge, this study is the first one investigating the diagnostic process of vulvodynia in general practice. Finally, the participants in the focus groups were heterogeneous with respect to gender, age, years of experience and practice type. Nevertheless, self-selection bias of our participants may be qualified as a limitation, as is seen as well in the low response ratio of approximately four per cent. As was shown in Table 1, most of our participants had an active role in the education of GPs and had a specific interest in vulvovaginal complaints. Perhaps, with GPs with an average interest in such complaints, other themes would have emerged.

### Barriers in discussing sexual issues

It has repeatedly been demonstrated that male as well as female GPs, encounter several barriers in discussing sexual issues [15–18]. Most barriers mentioned were lack of training, lack of time, embarrassment on the part of the GP, fear of opening ‘flood gates’, and having a different age or gender than the patient [15,16]. Training in communication skills and GPs’ personal attitude were found to be fundamental factors for sexual history taking and the management of sexual problems [17,18].

### Importance of gender in professional experience

Regarding importance of gender in professional experience, a small (*n* = 50) cross-sectional study using questionnaires found that more male than female GPs had little confidence in managing sexual problems in female patients, and, conversely, regarding male patients, confidence was lower in female GPs [16]. The same study showed that years of practice and the number of consultations in one week in which sexual problems were discussed, were protective factors for discussing sexual issues [16]. In another study, using a random sample of 512 family physicians and gynaecologists, physician gender concordance, increased years in practice, increased number of patients seen per week, and perceptions regarding practice experience, were found to be significant and independent predictors of increased confidence in treating patients with female sexual disorders [18]. A qualitative study among 22 GPs showed that according to these GPs patients preferred to see same-sex GPs regarding sexual health, and some GPs felt that as a consequence they became ‘de-skilled’ in dealing with sexual matters of opposite sex patients [19]. Finally, a small (*n* = 78) survey among a mixed group of physicians, showed that regarding sexual history taking, they perceived the greatest discomfort when interviewing opposite gender patients as well as very young and old-aged patients [20]. In short, findings concerning the relevance and influence of gender, age, experience and (lack of) training, are in line with our results. Apparently, same-sex matters.

### Professional uncertainty

Regarding the professional uncertainty GPs encounter during their consultations, their reactions appear to correspond to the coping strategy GPs use in general when confronted with patients with MUPS [21,22]. For example, the GPs in our study preferred to uphold the doctor–patient relationship by achieving mutual alliance characterized by ritual care (repeating regular tests or physical examination), as was described in a qualitative study among Dutch GPs [21]. Another qualitative study showed that the GPs used more emotion-focused approaches (aiming to reduce or manage the negative emotions caused by stress) instead of problem-focused strategies, indicating that they had difficulties in managing their stress when working with patients with MUPS [22]. The vignette we used in our study, a woman with recurrent vulvovaginal complaints, triggered some of the same reactions as patients with MUPS.

### Implications and future perspective

Our findings have implications particularly for the training of young male GPs and trainees. Male GP trainees should be better supported in the development of their attitudes and skills regarding taking a sexual history and performing a vulvovaginal examination. To a lesser extent, this also accounts for female GPs, as in most of the female GPs in this study symptoms associated with PVD were not known to them as well. Guidelines are currently available to support GPs in the management of PVD [23,24]. Moreover, training of GPs in coping with patients with MUPS may also help in coping with patients with PVD.

It is interesting to know whether the current management of vulvovaginal complaints by GPs likewise meets the needs and expectations of women. Insight into how patients with PVD perceive and evaluate management of their complaints by their GP may help to improve and facilitate both the diagnostic as well as the referral process of vulvovaginal complaints in general practice.

Finally, a quantitative survey would facilitate knowledge about the extent to which our findings are representative for all Dutch GPs. Unfortunately, quantitative research in general practice is limited, among other things, by a lack of clear classification of vulvodynia, as different ICPC codes could be applied. For future research in this field in general practice, well-defined coding of vulvodynia would be indispensable.

## Conclusions

Male gender, a lack of experience regarding PVD symptoms and negative emotional responses when faced with professional uncertainty, proved to be inhibiting factors, whereas female gender and the understanding that patients can profit from inquiring about sexual health issues were found to be facilitating factors.
